# Identifying the role of reactive oxygen species (ROSs) in *Fusarium solani* spores inactivation

**DOI:** 10.1186/s13568-016-0257-1

**Published:** 2016-10-01

**Authors:** Yilin Du, Houfeng Xiong, Shuangshi Dong, Jun Zhang, Dongmei Ma, Dandan Zhou

**Affiliations:** 1 Key Laboratory of Groundwater Resources and Environment, Ministry of Education, Jilin University, Changchun, 130021 Jilin China; 2 School of Environment, Northeast Normal University, Changchun, 130117 China; 3 Jilin Engineering Research Centre for Municipal Wastewater Treatment and Water Quality Protection, Changchun, 130117 China

**Keywords:** *F. solani*, Visible light, Photocatalysis, Inactivation, Soluble microbial products, Spore

## Abstract

The inactivation mechanism of photocatalytic disinfectants on bacteria is well known. In contrast, the potential inactivation of fungal spores by visible-light induced photocatalysis has been recognized, but the inactivation mechanism is poorly understood. We hypothesize that photocatalytically generated reactive oxygen species (ROSs) are directly involved in this mechanism. To test this hypothesis, we identified the roles of ROSs in the inactivation of *Fusarium solani* spores. As the photocatalysts, we doped TiO_2_ with 3 typical dopants, forming Ag/TiO_2_, N/TiO_2_ and Er^3+^:YAlO_3_/TiO_2_. The Ag/TiO_2_ photocatalysis was dominated by H_2_O_2_, with the longest lifetime among the investigated ROSs. Ag/TiO_2_ photocatalysis yielded almost 100 % inactivation efficiency and preserved the cell-wall shape of the spores, thus minimizing the biomolecule leakage. Er^3+^:YAlO_3_/TiO_2_ was dominated by h^+^ ROSs, yielding an inactivation efficiency of 91 %; however, the severe leakage released large numbers of molecular bio-products. Severe damage to the cell walls by the h^+^ species was confirmed in micrograph observations. Subsequent to cell wall breakage, the Er^3+^:YAlO_3_/TiO_2_ nanoparticles entered the spore cells and directly oxidized the intracellular material. The N/TiO_2_ photocatalysis, with •O_2_^−^ dominated ROSs, delivered intermediate performance. In conclusion, photocatalysts that generate H_2_O_2_-dominated ROSs are most preferred for spore inactivation.

## Introduction

According to the Food and Agriculture Organization of the United Nations, agriculture is the largest consumer of fresh water (Polo-López et al. 2014). Therefore, the reuse of wastewater for agriculture irrigation has received much attention in recent years. However, wastewater contains various pathogens that must be removed before the irrigation. Among these pathogens are phytopathogenic fungi such as *Fusarium*, *Pythium*, *Phytophthora* and *Olpidium* species. The *Fusarium* genus, which is widely distributed in water and soil systems, causes decay of roots, stems and flowers. *Fusarium* spores, which develop into *Fusarium* fungal cells, are very difficult to inactivate owing to their thick cell walls and strong environmental resistance (Polo-López et al. 2010).

One of the most promising methods for disinfecting *Fusarium* spores is photocatalytic inactivation (Fernández-Ibáñez et al. 2009). The photocatalytic inactivation should preferably be mediated by visible light, because UV light comprises only 5 % of the solar light (Zuo et al. 2012). Moreover, the photocatalytic performances of *Fusarium* spore disinfection are affected by the properties of the photocatalysts, such as the adsorption capability of the nanoparticles on the spores (Polo-López et al. 2010), and the morphology (nanotubes, nanoplates, nanorods or nanospheres) (Turki et al. 2013) and components (Fernández-Ibáñez et al. 2015) of the photocatalysts.

In essence, the inactivation capability of a photocatalyst depends on the reactive oxygen species (ROSs) it can generate. As photocatalysts adsorb irradiation with energies equal to or greater than their band gap energies, they generate e^−^/h^+^ pairs (Hou et al. 2012). In aqueous environments, ROSs are released through the chemical reaction of photogenerated species on TiO_2_ surfaces (Thabet et al. 2013). The released ROSs, which include e^−^, h^+^, •OH, H_2_O_2_, and •O_2_^−^, then disinfect the fungal spores (Xia et al. 2013). The significant role of ROSs in phototocatalytic bacterial disinfection is well recognized (Foster et al. 2011). The inactivation efficiency against *Escherichia coli* is linearly related to the steady-state •OH concentration, suggesting that •OH is the predominant inactivation species in this organism (Cho et al. 2005). This conclusion was disputed by Kikuchi et al. (1997), who separated *E*. *coli* suspension from a TiO_2_ thin film through hydrophilic polytetrafluoroethylene membranes. They found that neither the half-life nor the half diffusion length of •OH was sufficient to traverse the membrane and inhibit the *E*. *coli* cells. Therefore, they concluded that the major bactericidal species was H_2_O_2_ rather than •OH. ROSs can also attack the cell walls, causing leakage of intracellular macromolecules such as proteins and nucleic acids from the cells (Rahmanto et al. 2005).The leaking biomolecules can lower the efficiency of the treatment and adversely affect the effluent quality, seriously compromising the health of the receiving waters (Xie et al. 2012). Consequently, when applying a photocatalytic disinfection, we must also monitor the subsequent increment in soluble microbial products (SMPs).

The effects of ROSs on the disinfection efficiency and SMP release are well known in bacterial disinfection, but are poorly understood in fungal spore disinfection. In visible light photocatalysis, modifying the TiO_2_ surface with different dopants will alter the generated ROSs (Chong et al. 2014). The diverse TiO_2_ crystal structure admits a variety of dopants (Shah et al. 2002), inducing different spore-inactivation mechanisms. Among the versatile dopants trialed in recent studies, the most effective were the metallic element Ag (Lee et al. 2005), the nonmetal element N (Nakamura et al. 2004) and the upconversion luminescence agent Er^3+^:YAlO_3_ (Zhou et al. 2015). Under visible light, the silver atoms in Ag/TiO_2_ play a co-catalytic role by injecting plasmon-induced electrons into the photocatalyst. Thus, TiO_2_ generates high e^−^/h^+^ pair separation under visible light (Lee et al. 2005). In contrast, the nitrogen-doped TiO_2_ can respond to visible light because the N atoms decrease the band energy gap (Lu et al. 2014). In Er^3+^:YAlO_3_/TiO_2_, the photocatalytic material assembles with the upconversion luminescence agents Er ^3+^:YAlO_3_ (Wang et al. 2010), transforming the visible light to UV light and thus satisfying the requirement for anatase TiO_2_ photocatalysis (Dong et al. 2015).

In summary, photocatalytic inactivation of fungal spores is worthy of consideration, but the activation mechanism remains to be elucidated. In this work, we prepared three kinds of photocatalysts; a metal-doped photocatalyst (Ag/TiO_2_), a nonmetal-doped photocatalyst (N/TiO_2_) and an upconversion luminescence agent-doped photocatalyst (Er^3+^:YAlO_3_/TiO_2_), which were expected to generate different ROSs. We investigated the ability of the three photocatalysts to inactivate *Fusarium solani* (*F. solani*) spores, and the subsequently retained inactivation by-products. By analyzing the dominant ROSs released by the photocatalysts, we identified the preliminary mechanisms of photocatalytic spore inactivation, and highlighted the need for appropriate photocatalysts selection in future.

## Materials and methods

### Catalyst preparation

Nano-sized Ag/TiO_2_ particles were prepared by a sol–gel process as described in Lee et al. (2005). The nitrogen-doped photocatalysts were prepared by the calcinations method, using urea as the nitrogen source (Kontos et al. 2008). The Er^3+^:YAlO_3_/TiO_2_ was prepared by a sol–gel method described in Dong et al. (2015).

### Fungal strain, cultivation and enumeration

*Fusarium solani* was purchased from Beina Chuanglian Biotechnology Institute. (Beijing, China, No. BNCC144579). Colonies of *F. solani* were transferred to sporulation agar medium containing potassium chloride and kept at 25 °C for 15 days (Fernández-Ibáñez et al. 2009). The spores were washed from the medium using sterilized distilled water. The obtained mixed suspension was centrifuged at 300×*g* for 10 min and washed three times with sterilized distilled water. The concentration of the suspension was determined by direct count in a counting chamber (Turki et al. 2013). The initial experimental concentration was approximately 10^4^ CFU/mL. The fungal spore concentration during photocatalysis was determined by the pour plate technique (i.e., by spreading 100 µL of the sample in the plate, and counting the resulting colonies) (Polo-López et al. 2014). All samples and treatments were prepared in triplicate and the plates were incubated at 28 °C for 2 days in the dark before counting (Polo-López et al. 2014).

### Photocatalytic inactivation

The experiments were performed in 27.5-mm × 95-mm (diameter × height) sealed glass bottles with a working volume of 40 mL. The light source was a 300-W xenon lamp with a 420-nm cutoff and an intensity of ~100 mW/cm^2^. The light beam was horizontally projected on the side wall of the bottle. In a typical inactivation test, the bottles containing magnetic stirrers were pre-sterilized before each batch.

The *F. solani* spores (initial concentration ~ 10^4^ CFU/mL) and photocatalyst powder (1 g/L) were then added to the sterilized bottles. All experiments were performed in triplicate at room temperature (~20 °C).

The scavenging species and their concentrations in the scavenging experiments were as follows: 0.5 mmol/L sodium oxalate, 0.5 mmol/L isopropanol, 0.05 mmol/L Cr(VI), 0.1 mmol/L Fe(II)-EDTA, and 2 mmol/L TEMPOL. By quenching the specific reactive species with individual scavengers, we can specify the reactive species’ contributions in the different photocatalytic systems: Cr(VI) for e^−^, isopropanol for •OH, sodium oxalate for h^+^, Fe(II) for H_2_O_2_, and TEMPOL for •O_2_^−^ (Xia et al. 2013).

### Analytical methods

#### X-ray diffraction (XRD) analysis

Powder XRD patterns were acquired by a Bruker D8 Advanced X-ray diffractometer using Cu Kα radiation (λ = 0.15418 nm) at a scanning rate of 2°/min. The 2*θ* range was 10°–90°.

#### Optical microscope

Drop samples were collected from the reaction solution at 0 and 4 h. The samples were observed under an optical microscope (CKX41, Olympus).

#### Transmission electron microscopy (TEM)

Samples (10 mL) were collected from the reaction solutions at 0 and 4 h, centrifuged and washed three times with 0.1 M phosphate buffer (PBS, pH = 7.2). Cell pellets of the samples were prepared by pre-fixing in 2.5 % glutaraldehyde at 4 °C for 12 h, washing three times with PBS, then pre-fixing in 1 % osmium tetroxide at 4 °C for 3 h. The cell pellets were dehydrated through a series of ethanol with graded concentration and embedded in Spurr solution at 70 °C for polymerization. The polymerized samples were sectioned into ultrathin slices (70 nm) using an ultramicrotome (Leica, Reichert Ultracuts, Wien, Austria), and stained with uranyl acetate and lead citrate. Finally, the sections were observed in a JEM1230 transmission electron microscope (JEOL Ltd., Tokyo, Japan).

#### Three-dimensional fluorescence spectra (EEM) measurements

The reaction solution (10 mL) was sampled from the three photocatalyst preparations at 0, 1 and 7 h, then filtered through a 0.22-μm membrane to remove the fungal cells. EEM spectroscopy measurements were conducted using a fluorescence spectrometer (F-7000, Hitachi, Japan) at a scan rate of 2000 nm/min and an excitation/emission slit bandwidth of 5 nm. The scanning field was set to 220–600 nm for the emission spectra and 220–450 nm for the excitation spectra.

#### Molecular weight (MW) analysis

The molecular weight of the aqueous phase was analyzed by gel filtration chromatography (GFC, Shimadzu, Japan), which performs high performance liquid chromatography (LC-10A, Shimadzu, Japan) with a differential refractive index (RID-10A, Shimadzu, Japan) detector. As the standard, we used glucan, which covers a molecular weight range of 200–1500,000 Da. The column temperature was controlled at 40 °C. The mobile phase was deionized doubly distilled water with a flow rate of 0.6 mL/min, and the hydraulic detention time of the column was 30 min.

## Results

### Characterization of the prepared photocatalysts

**Fig. 1:**
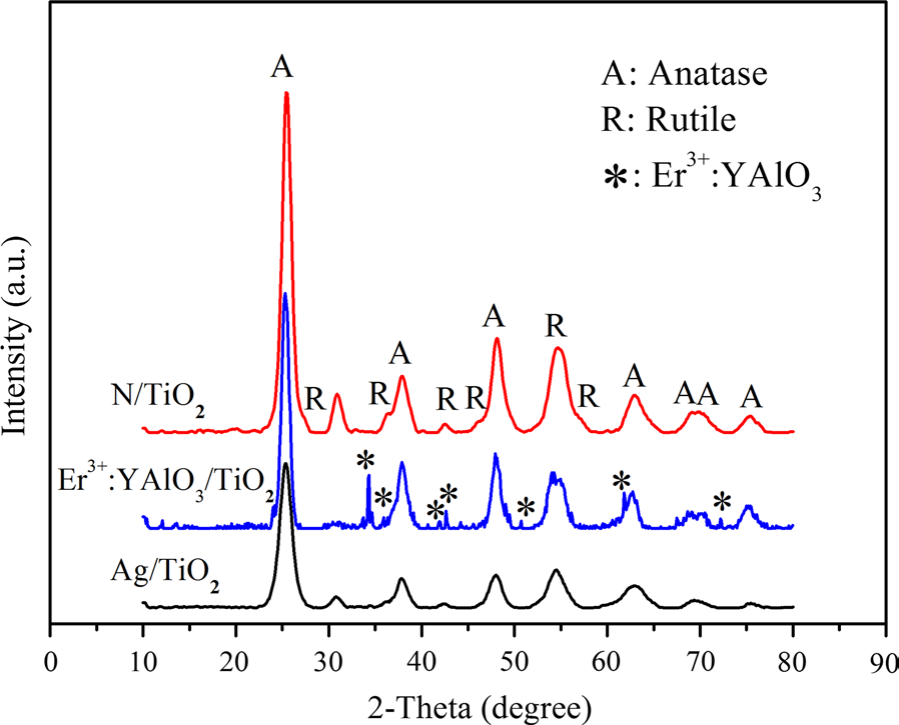
XRD spectra of the prepared photocatalysts

Figure 1 shows the XRD spectra of the prepared N/TiO_2_, Er^3+^:YAlO_3_/TiO_2_ and Ag/TiO_2_ photocatalysts. The peaks in the XRD spectra of the three photocatalysts are generally sharp, with small full-width-at-half maximums. Therefore, the formation of the crystals was basically complete, and the photocatalysts were receptive to the photocatalytic reaction. The doping mechanisms of these nanoparticles were described above.

The three photocatalysts presented different characteristics as they were doped with different materials. The phase structure of the prepared Ag/TiO_2_ was pure anatase TiO_2_. The N/TiO_2_ nanoparticles exhibited a phase structure of mixed anatase and rutile with mass fractions of ~97 and 3 %, respectively (computed by the Spurr–Myers equation; Spurr and Myers 1957). Er^3+^:YAlO_3_/TiO_2_ presented two crystal forms; the visible light up-conversion components Er^3+^:YAlO_3_ and anatase, indicating that Er^3+^:YAlO_3_ was successively doped in the anatase TiO_2_ (Dong et al. 2015).

### Inactivation of *F. solani* spore by the prepared photocatalysts

**Fig. 2:**
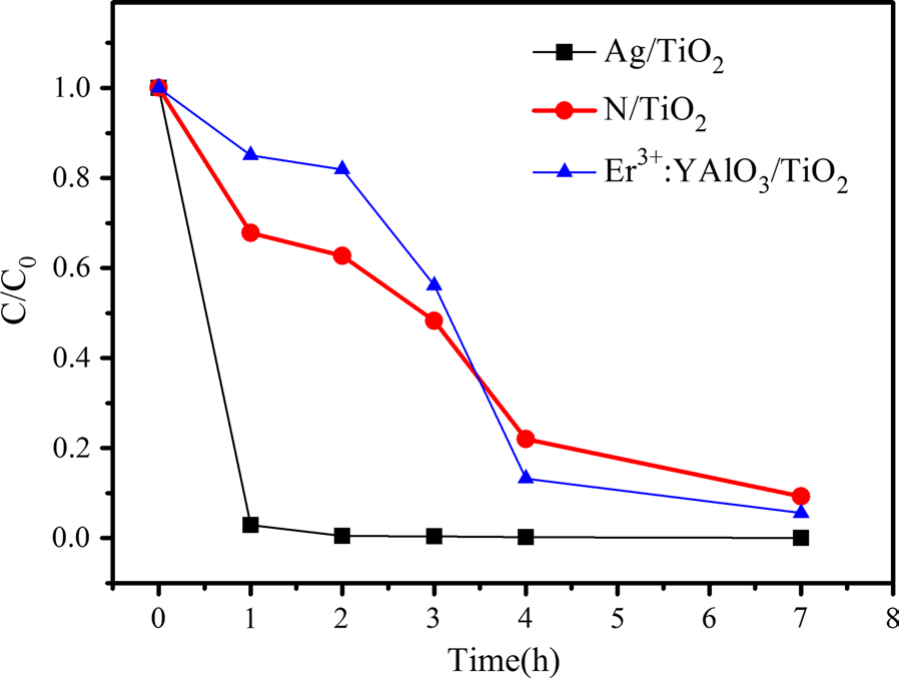
Inactivation of *F. solani* using Ag/TiO_2_, N/TiO_2_ and Er^3+^:YAlO_3_/TiO_2_photocatalysts under visible light irradiation. All the values are obtained based on the average of three times experiments

Figure 2 compares the *F. solani* spore counts during photocatalysis inactivation with the three prepared photocatalysts under visible light irradiation. All of the photocatalysts efficiently inactivated the spores. Over a 4-h period, N/TiO_2_ and Er^3+^:YAlO_3_/TiO_2_ (at dosages of 1 g nanoparticles/L) achieved inactivation efficiencies of 78 and 87 %, respectively. During the following 3 h, the removal rates of living cells continuously increased, reaching 91 and 94.3 %, respectively. Notably, the Ag/TiO_2_ nanoparticles inactivated 99.5 % of the *F. solani* cells within 1 h of irradiation, and nearly all cells were inactivated within 4 h. Obviously, Ag/TiO_2_ demonstrated higher fungal infection capability than the other photocatalysts. In comparison, none of the control treatments—visible light photolysis alone (no catalysts), dark adsorption (no irradiation) and the blank control (neither catalysts nor irradiation)—noticeably inactivated the *F. solani* spores (data not shown).

### Micro-observation of the inactivated spores

**Fig. 3:**
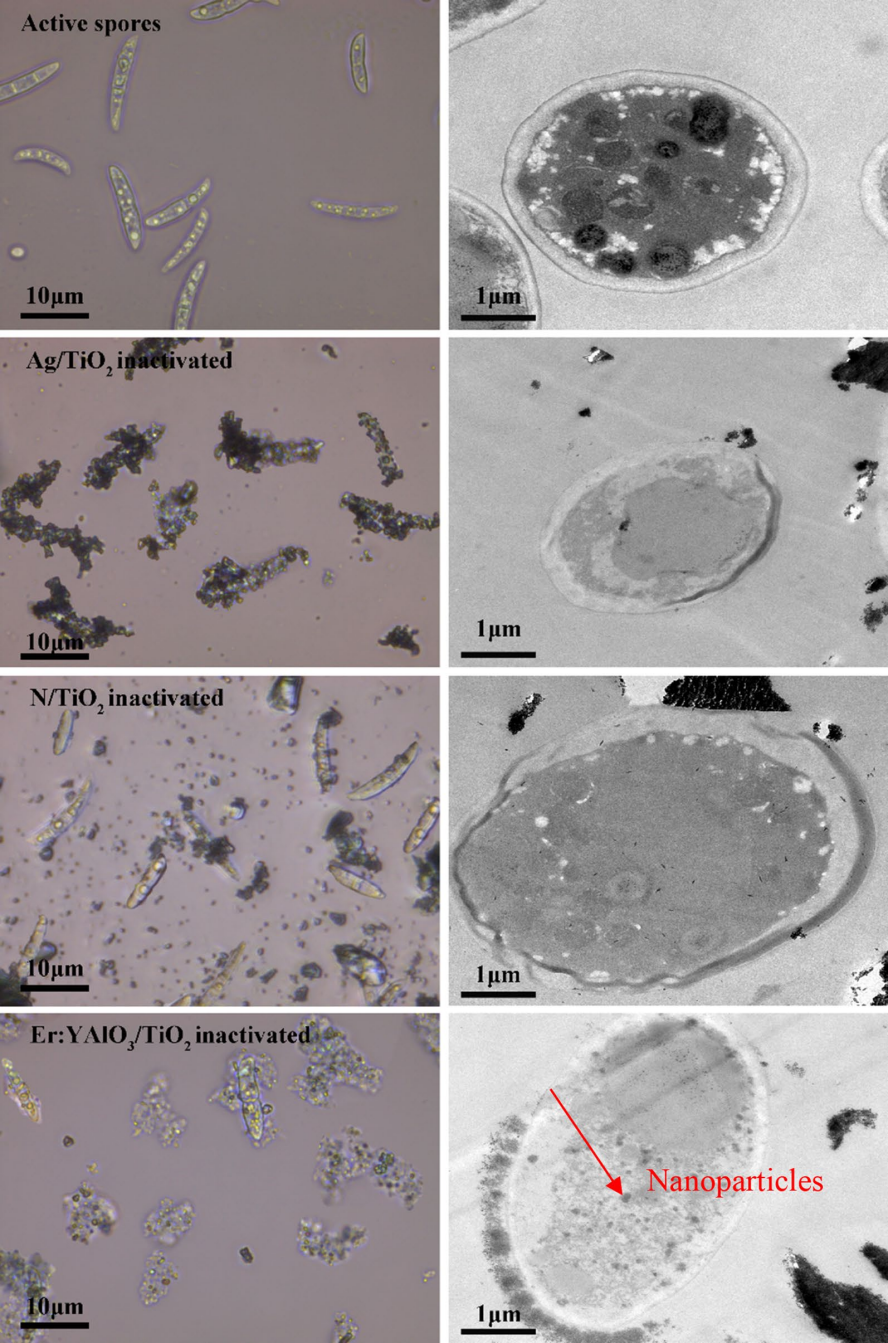
Optical microscope images (*left column*) and TEM (*right column*) of the active. *F. solani*, and photocatalytically treated *F. solani* with Ag/TiO_2_, N/TiO_2_ and Er^3+^:YAlO_3_/TiO_2_, respectively, after 4 h visible light irradiation. The *red arrow* pointed the Er^3+^:YAlO_3_/TiO_2_ photocatalysis which entered the spores

To better understand the inactivation mechanism, the outer shape and inner-microstructures of *F. solani* before and after photocatalysis were examined under an optical microscope and TEM, respectively. As shown in Fig. 3, the active *F. solani* spore cells were sickle-shaped, with approximate lengths of 10 µm and cell-wall thicknesses of 0.3 μm. Regular cell walls, membranes, nuclei and mitochondria-like components were clearly visible inside the cells.

All of the inactivation protocols shrunk the cells, deformed their shapes, and generated large quantities of cell debris. The photocatalyst nanoparticles were obviously adsorbed on the cell surfaces, causing initial physical damage to the cells (Caballero et al. 2009). The Ag/TiO_2_ photocatalysis, which performed most efficiently among the three treatments, exhibited the highest number of photocatalysts enclosing the spores. Such direct contact suggests direct oxidation of the cell components and high killing efficiency against the microorganisms (Rahmanto et al. 2005), and is especially significant for *F. solani* spores with their relatively thick cell walls (Thabet et al. 2014). Interestingly, the Er^3+^:YAlO_3_/TiO_2_ protocol performed less efficiently than Ag/TiO_2_, but caused much greater spore damage and produced the most cell debris among the protocols. These various consequences were related to the different ROSs characteristics of the photocatalysts.

The TEM results revealed microscopic changes in the spore cells (Fig. 3). After 4 h photocatalysis by all three protocols, the cells were disrupted to various degrees, showing damage features such as plasmolysis and ghosting of the intracellular vacuoles. Most of the intracellular components became unclear, indicating their decay. The Ag/TiO_2_ protocol best preserved the cell wall structure, but cytoplasm leaking was observed. The N/TiO_2_ protocol partially oxidized the cell wall, releasing the spore cell contents; further photocatalytic oxidization should completely kill the spores. No nanoparticles were observed in the spore cells subjected to these two protocols. In contrast, the Er^3+^:YAlO_3_/TiO_2_ photocatalysis introduced some nanoparticles into the inactivated spores (indicated by the red arrow). In these spore cells, the cytoplasm was seriously disrupted and even partially disappeared. The Er^3+^:YAlO_3_/TiO_2_ treatment yielded the thinnest cell walls among the three protocols, suggesting the strongest attacking capability on the spore cell wall, despite its weaker inactivation capability than Ag/TiO_2_ (see Fig. 2).

### Biomolecules leakage and degradation during inactivation

**Fig. 4:**
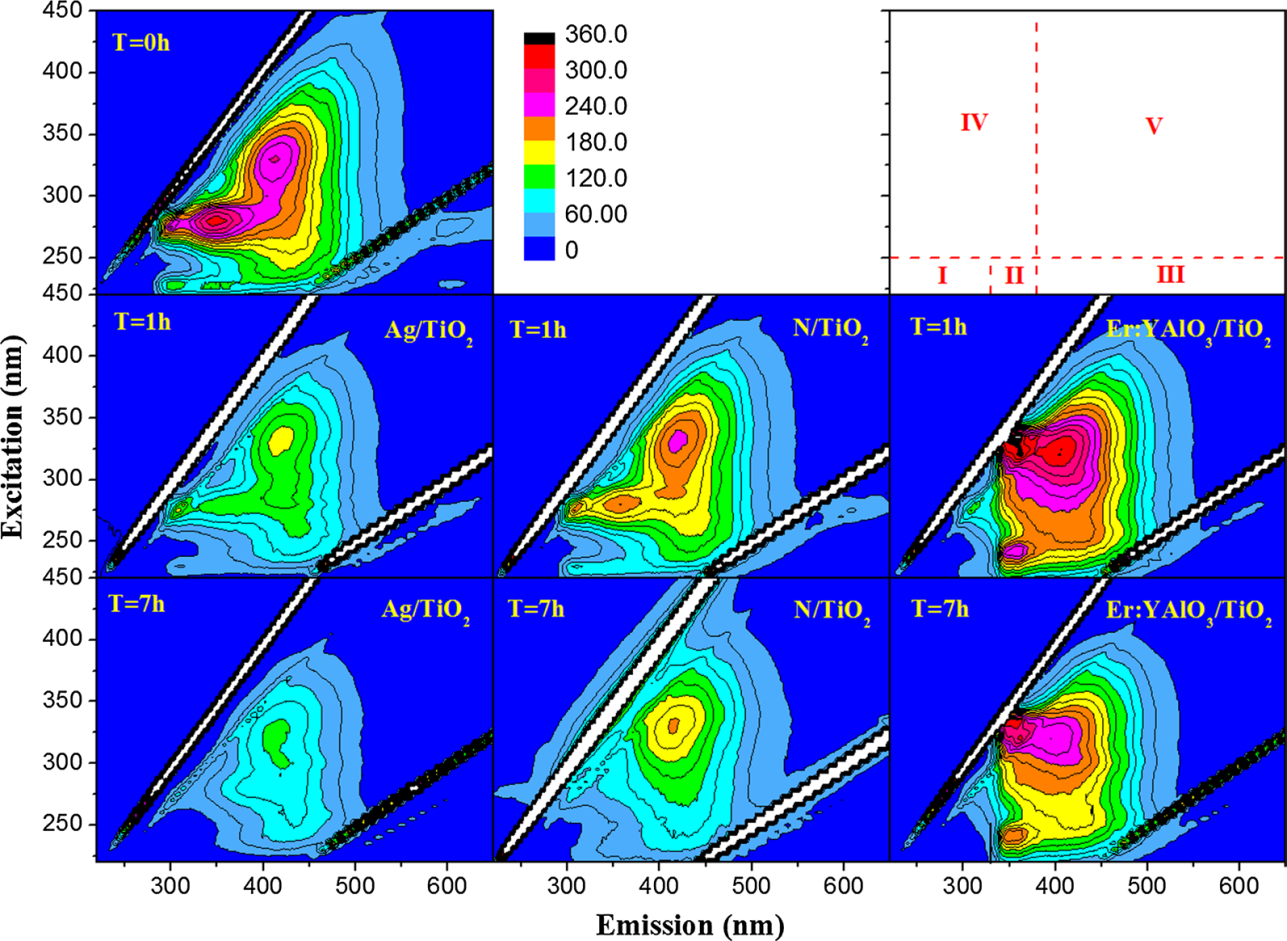
EEM spectra of the soluble microbial products in the bulk water after *F. solani* inactivation. Five regions were classified, peaks at region I (Ex/Em = 220–250/280–320) are related to tyrosine/tryptophan amino acid; region II is tyrosine/tryptophan protein region with Ex/Em = 220–250/320–380; region III is fulvic acid region with Ex/Em = 220–250/380–460; region IV is soluble microbial byproduct-like material with Ex/Em = 250–380/>280–380 and region V is humic acid-like organics with Ex/Em = 250–380/380–450

The EEM spectrum provides clues into the subsequent SMP release during the photocatalytic inactivation. Figure 4 shows the main SMP constituents in the spore cells prior to inactivation. We observe tyrosine/tryptophan amino acids (in region I), soluble microbial byproduct-like materials (in region VI) and humic acid-like organics (in region V). As evidenced by the gradual weakening of their peaks, all of these SMP constituents were significantly reduced by the Ag/TiO_2_ and N/TiO_2_ photocatalysts. Again, the Ag/TiO_2_ photocatalysis demonstrated much higher removal efficiency for the generated SMPs than the other protocols. The tyrosine/tryptophan amino acid and soluble microbial byproduct-like compounds were almost completely removed, and the humic acid-like compounds were dramatically decreased. It appears that the Ag/TiO_2_ and N/TiO_2_ treatments simultaneously degraded the leaked cell contents (confirmed by the TEM results) while inactivating the *F. solani* cells. In the EEM spectra of the Er^3+^:YAlO_3_/TiO_2_ protocol, the peaks of the tyrosine/tryptophan proteins and soluble microbial byproduct-like compounds were red shifted and enhanced after 1 h, indicating that additional molecules of these species had formed during the inactivation. Moreover, these peaks were only slightly weaker after 7 h treatment, confirming that the new compounds were retained.

**Fig. 5:**
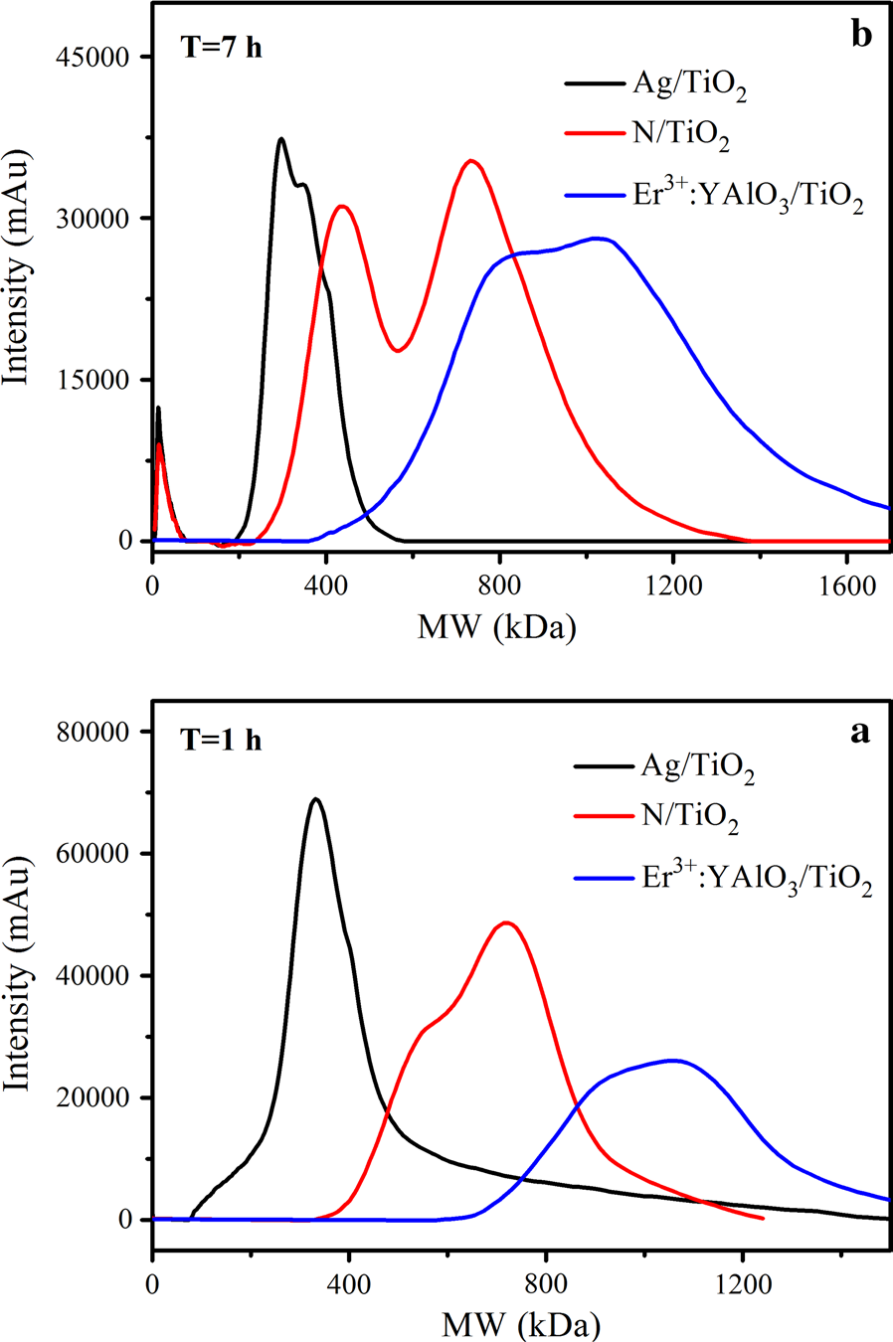
SEC chromatograms of SMPs during photocatalysis inactivation 1 h (**a**) and 7 h (**b**)

The above discussion of the SMP components was supported by the size exclusion chromatography and high performance liquid chromatography (SEC-HPLC) results (see Fig. 5). During 1 h inactivation, the molecular weights (MWs) of the SMPs decreased from 400–1500 kDa to 100–450 kDa in the Ag/TiO_2_ treatment and to 340–1200 kDa in the N/TiO_2_ treatment. Ag/TiO_2_ destroyed the initial cellular macromolecules more extensively than N/TiO_2_, and ultimately degraded them to much smaller and presumably less harmful compounds (Ni et al. 2010). After 7 h inactivation, intermediates with MWs as small as 10–70 kDa were detected in the Ag/TiO_2_ and N/TiO_2_ inactivation protocols, and the intensities of heavier intermediates had significantly decreased. In contrast, the Er^3+^:YAlO_3_/TiO_2_ protocol slightly increased the MW distribution of the SMPs, from its initial 400–1500 kDa to 600–1600 kDa after 1 h. The increase is attributable to the large MWs of the leaked intercellular material. The intensities of the accumulated SMPs in this MW range increased after 7 h, indicating their harmful and photocatalytically non-degradable characteristics. These high-weight SMPs (400–1600 kDa) are potentially harmful and persistent in the environment (Jarusutthirak and Amy 2007).

## Discussion

In this work, *F. solani* spores were inactivated by three prepared photocatalysts, and different inactivation efficiencies, cell damage mechanisms and subsequent SMP leakage levels were observed. The Ag/TiO_2_ can kill the spores while maintaining the integrity of their cell walls and simultaneously degrading the SMPs leaked throughout the inactivation. The N/TiO_2_ nanoparticles ruptured the cell walls but similarly degraded the leaked SMPs. However, the Er^3+^:YAlO_3_/TiO_2_ nanoparticles entered the spore cells and significantly oxidized the intercellular contents, which likely explains the large molecular weights of the released SMPs. These differences among the inactivation behaviors were determined by the ROS species generated by the photocatalysts under visible light irradiation (Sun et al. 2014). The photogenerated ROSs, including h^+^, •OH, H_2_O_2_, and •O_2_^−^, are all responsible for bacterial inactivation and play different roles in various photocatalytic systems (Hou et al. 2012).

**Fig. 6:**
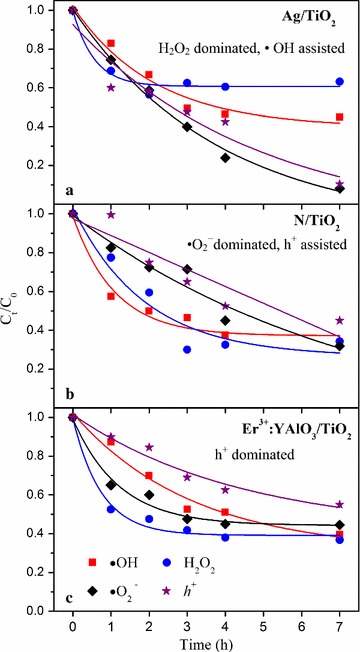
Inactivation efficiency of *F. solani* by Ag/TiO_2_, N/TiO_2_ and Er^3+^:YAlO_3_/TiO_2_ photocatalysts with different scavengers. Legends are the scavenged ROS, and all the values are obtained based on the average of three times experiments

As shown in Fig. 6, the Ag/TiO_2_ photocatalysis system was dominated by H_2_O_2_ and assisted by •OH. In the N/TiO_2_ system, h^+^ and •O_2_^−^ played the important roles, with •O_2_^−^ being the most determining species. The Er^3+^:YAlO_3_/TiO_2_ inactivation system was dominated by h^+^. These three ROSs have different average life-spans (4 × 10^−9^ and 2 × 10^−10^ s for •OH and •O_2_^−^, respectively, and even shorter for h^+^) (Xia et al. 2013). Conversely, H_2_O_2_ is relatively stable with a half-life of several days in water (Xia et al. 2013). Moreover, in the following generation sequence of ROSs (Chatterjee and Dasgupta 2005), ROSs produced in the earlier steps possess higher oxidizing potential.

TiO_2_ + *hν* → e^−^ + h^+^

h^+^ + H_2_O → •OH + H^+^

h^+^ + OH^−^→ •OH

O_2_ + e^−^ → •O_2_^−^

•O_2_^−^ + H^+^ → HO_2_•

2 HO_2_• → O_2_ + H_2_O_2_

H_2_O_2_ + O_2_^−^ → •OH + OH^−^ + O_2_

Thus, the oxidizing potentials of the dominant ROSs generated in this work increased in the order h^+^ > •O_2_^−^ > H_2_O_2_.

Accordingly, H_2_O_2_ has the longest span life but the weakest oxidization capability among the three ROSs. This species, which dominated the Ag/TiO_2_ photocatalysis, diffused into the cells without seriously damaging their cell structures. Consequently, the H_2_O_2_ molecules permeated the cell membranes, damaging the spores’ interiors without excessively leaking their cell contents (as confirmed by the EEM and SEC results). However, the dominant ROS in Er^3+^:YAlO_3_/TiO_2_ photocatalysis was h^+^, with the strongest oxidative potential but shortest life-span among the three ROSs. This species was generally produced on the surface of the catalyst and directly attacked the cell wall. The Er^3+^:YAlO_3_/TiO_2_ nanoparticles then entered the cells through the broken cell wall, causing abundant leakage of the intracellular components and subsequent pollution of the bulk water quality. The N/TiO_2_ photocatalysis was dominated by •O_2_^−^, with a life-span and oxidization capability between those of H_2_O_2_ and h^+^; consequently, the anoxidation performance of this photocatalysis was intermediate between Ag/TiO_2_ and Er^3+^:YAlO_3_/TiO_2_. In summary, the H_2_O_2_-dominated Ag/TiO_2_ photocatalysis delivered outstanding performance in both inactivation efficiency and SMP-leakage control.

## Abbreviations

ROSs: reactive oxygen species
*F. solani*: *Fusarium solani*
SMPs: soluble microbial products
XRD: X-ray diffraction
TEM: transmission electron microscopy
EEM: three-dimensional fluorescence spectra
MW: molecular weight
SEC: size exclusion chromatography and
HPLC: high performance liquid chromatography
